# Simultaneous treatment of intracranial complications of paranasal sinusitis

**DOI:** 10.1007/s00405-018-4932-5

**Published:** 2018-03-13

**Authors:** Witold Szyfter, Anna Bartochowska, Łukasz Borucki, Adrian Maciejewski, Aleksandra Kruk-Zagajewska

**Affiliations:** 1 Department of Otolaryngology and Laryngological Oncology, University of Medical Sciences, Przybyszewskiego Street 49, 60-355 Poznań, Poland; 2 Department of Rescue Medicine, University of Medical Sciences, Poznań, Poland

**Keywords:** Sinusitis, Brain abscess, Meningitis, Empyema, Intracranial complication

## Abstract

**Purpose:**

The objective of this study was to analyse 51 patients with intracranial complications of sinusitis treated in the Department of Otolaryngology and Laryngeal Oncology at Poznań University of Medical Sciences from 1964 to 2016.

**Materials and methods:**

Males made up a significant portion of study participants at 70.5%. Treatment included simultaneous removal of inflammatory focal points in the paranasal sinuses and drainage of cerebral and epidural abscesses and subdural empyemas under the control of neuronavigation preceded by the implementation of broad-spectrum antibiotics continuously for 4 weeks. Seventy-three intracranial complications were found among 51 patients. Of the 51 patients, 25 had frontal lobe abscesses (including multiple abscesses). Other complications included the following: 16 epidural abscesses, 9 subdural empyemas, 15 meningitis cases, 3 intracerebral abscesses, 3 sinus thrombosis cases and 2 patients with cerebritis. Co-occurrence of these complications worsened the state of the patient and increased the duration of treatment. Patients with frontal lobe abscesses had a better prognosis and less pronounced neurological symptoms in recent years versus earlier treatment approaches.

**Conclusions:**

Simultaneous treatment of intracranial complications of sinusitis is an effective treatment method that has minimal burden for the patient. From 1964 to 1978, three deaths (17%) were reported among patients with these complications. Since 1978, no deaths were reported in the clinic.

## Introduction

Acute and chronic sinusitis is a common medical condition that may result in intracranial, extracranial or systemic complications. Of these complications, intracranial complications rarely occur [1]. The incidence of these complications varies depending on geographical region, climate and socioeconomic stratification, which appears more often in developing countries [2, 3]. In the literature, there is a lack of representative data on the occurrence of these phenomena in the larger scope. Usually, only data fragments are published [2–6].

The diverse array of symptoms associated with these complications during the onset of disease and the extensive use of antibiotics prior to delivering appropriate therapeutic measures can often mask significant symptoms of intracranial complications [7]. This leads to a difficult and complex clinical manifestation (with regard to diagnosis and treatment) that may directly threaten the patient’s life and require prompt and definitive surgical treatment [8–10]. The purpose of this study was to present our results on the management of intracranial complications of sinusitis, specify the clinical manifestations of the pathologies and to propose an adequate management strategy. The treatment was performed in the Department of Otolaryngology at Poznań University of Medical Sciences from 1964 to 2016.

## Materials and methods

The design of this study was to retrospectively analyse the results of 51 patients with intracranial complications of sinusitis that were admitted to the Department of Otolaryngology and Laryngeal Oncology at Poznań University of Medical Sciences during a 53-year period between 1964 and 2016. The paper does not include an analysis of complications manifesting from other origins, e.g. ocular, haemorrhagic or traumatic. The patient notes were collected and each patient’s data set was thoroughly evaluated for age, gender, history of present illness, diagnostic workup including imaging studies and results of microbiological cultures (from paranasal sinuses, epidural and cerebral abscesses, subdural empyemas and cerebrospinal fluid), therapeutic measures (past surgeries or antibiotic treatment) and documentation from outpatient visits. Additionally, in-depth physical examinations were performed paying special attention to otolaryngological, neurological and ophthalmological aberrations.

Due to the long observation period of 53 years, significant technological improvements were made in diagnostic radiology exams. The study population was, therefore, divided into three periods: 1964–1978, 1979–1999 and 2000–2016 (Table 1).

**Table 1:** Diagnostic methods, treatment and mortality analysed in respective time periods

	1964–1978	1979–1999	2000–2016
Diagnostic method	Physical examination Radiological imaging Sinus Cranium Neck angiography Cerebral cortex CT cranium Fundus examination EEG	CT/MRI of the cranium	CT/MRI of the cranium
	Consultation with ophthalmologist, neurologist and neurosurgeon		
Treatment methods	Opening the sinuses externally Puncture/drainage of the abscess (Zakrzewski method) Crystallised penicillin + metronidazole	Opening the sinuses externally Puncture/drainage of the abscess (Zakrzewski method) Crystallised penicillin + metronidazole/cephalosporin III generation	Opening the sinuses externally/via endoscopy Puncture/drainage of the abscess (Zakrzewski method) NEURONAVIGATION Piperacillin tazobactam/carbapenem
Mortality	17%	0%	0%

During the first period (1964–1978), paranasal sinusitis was recognised by radiological summation images of the sinuses. Investigation for intracranial complications of sinusitis (e.g. an evaluation for cerebral abscesses) was extremely difficult and required a differentiation technique that would compare the abscess to other pathological changes in the central nervous system, e.g. brain tumour. Therefore, the examination for the suspicion of cerebral abscess was based on subjective opinions, clinical picture and carotid angiography. Carotid angiography was used because vascular silence could be observed at the location of the cerebral abscess because the arteries did not dilate. Carotid angiography was performed only in the anterior and posterior views for intracranial complications of sinusitis. In the anteroposterior projection, a significant degree of arcuate displacement of the coronary artery towards the right side could be observed. In the lateral view, the frontal artery was displaced posteriorly and stenosis was observed in the main artery with its branches leading to an expanding intracerebral mass [11]. Ophthalmological examination, electroencephalogram and radiographic imaging of the cranium and cerebellum were also examined during this period.

During the second period (1979–1999), both paranasal sinusitis and intracranial complications were diagnosed using computer tomography (CT). This method allowed for the precise location of inflammatory changes in the sinuses [12]. The CT was able to accurately assess location, shape, cerebral and/or multiple abscesses, epidural abscess, subdural empyema and the area surrounding cerebral oedema with coexisting ventricular displacement and also monitor the efficacy of the treatment.

Magnetic resonance imaging (MRI) was used for diagnosing intracranial complications of sinusitis during the last period of the study (2000–2016). From 2007 onwards, MRI-based neuronavigation systems have been used to diagnose and treat cerebral abscesses at the clinic. Neuronavigation allowed for precise localisation of pathognomonic structures and aided in the evacuation of purulent intracranial lesions [7].

## Results

Fifty-one patients with intracranial complications of sinusitis were included in the study from 1964 to 2016. The participants were made up of 36 males and 15 females with a median age of 39 years (range 12–72). There was an increased prevalence of patients aged 24–25 years. The study population was divided into three groups depending on the different treatment modalities available during the previously mentioned time periods: 16 patients in the first period (1964–1978), 9 patients in the second period (1979–1999) and 26 in the last period (2000–2016).

**Fig. 1:**
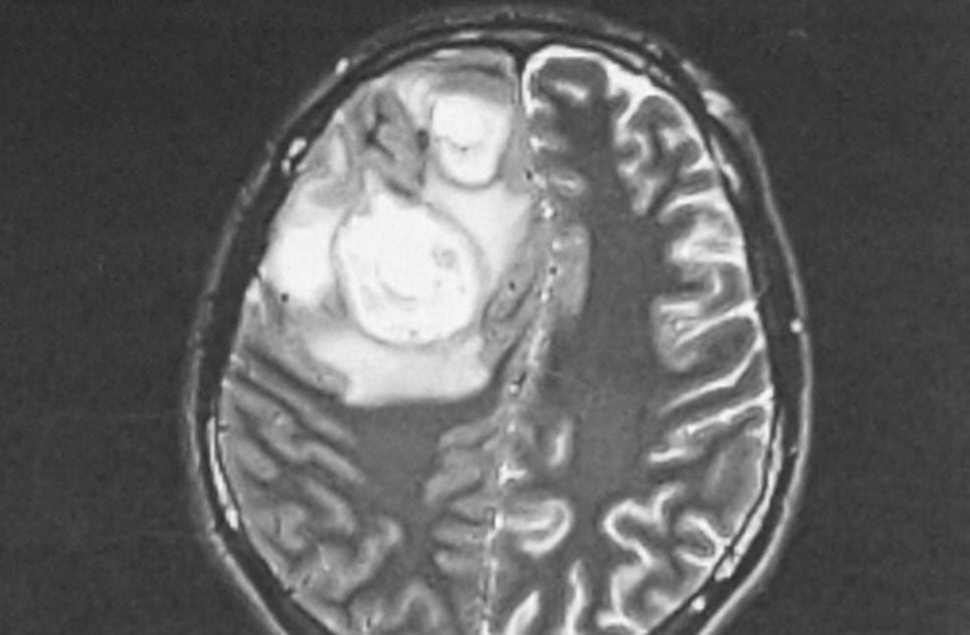
MRI of the cranium in a 28-year-old man. Two abscesses with diameters of 45 and 28 mm in the right frontal lobe. There is oedema of the brain tissue and thickened cerebrospinal fluid over the area where the abscess is located. The mass is clearly visualised and compression and displacement of the right chamber are also noted

**Fig. 2:**
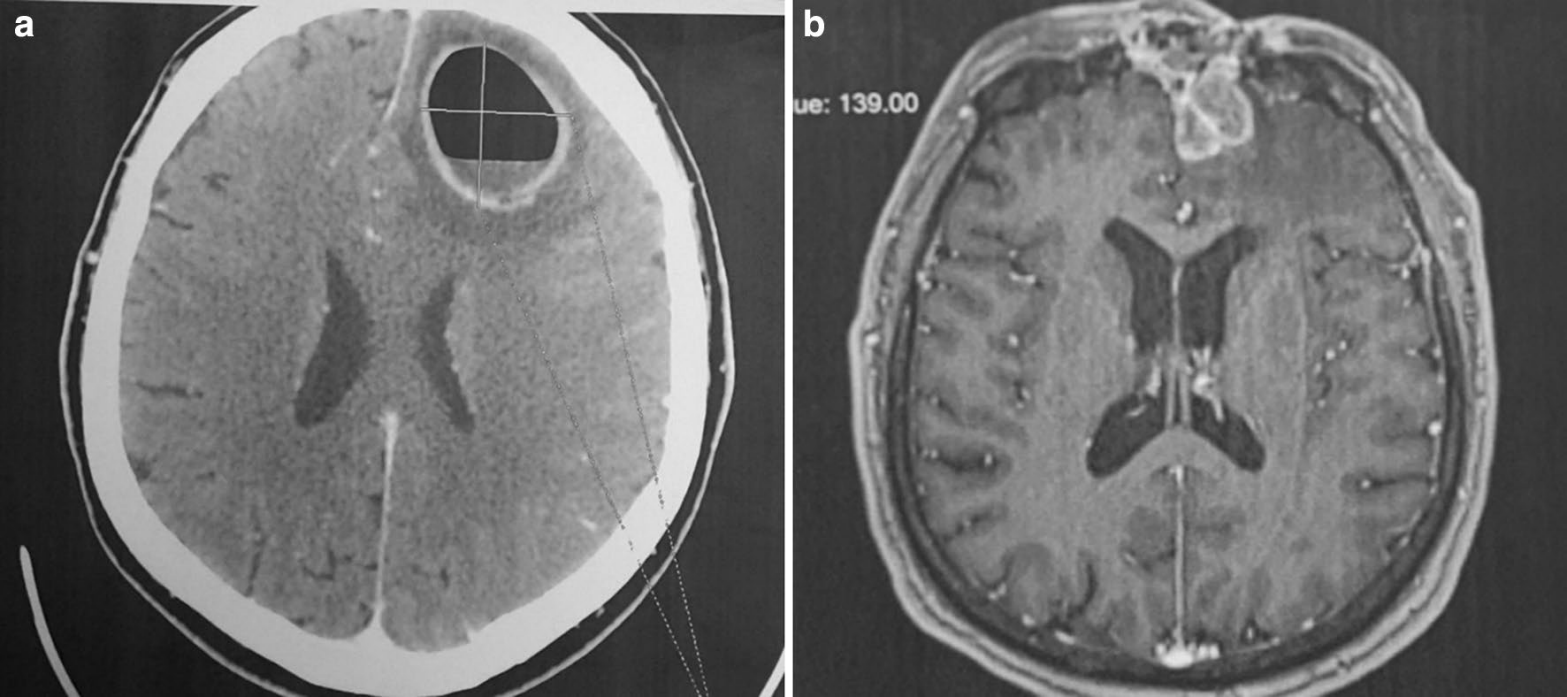
**a** MRI of the cranium in a 58-year-old man. The abscess with a distinct size of 38 × 28 mm in the left frontal lobe produces a marked displacement and compression of the left chamber. **b** MRI of the cranium in the same patient hospitalised after 12 months due to the recurrence of symptoms. Irregular multichamber abscess in the left frontal lobe measuring 32 × 24 × 16 mm

**Fig. 3:**
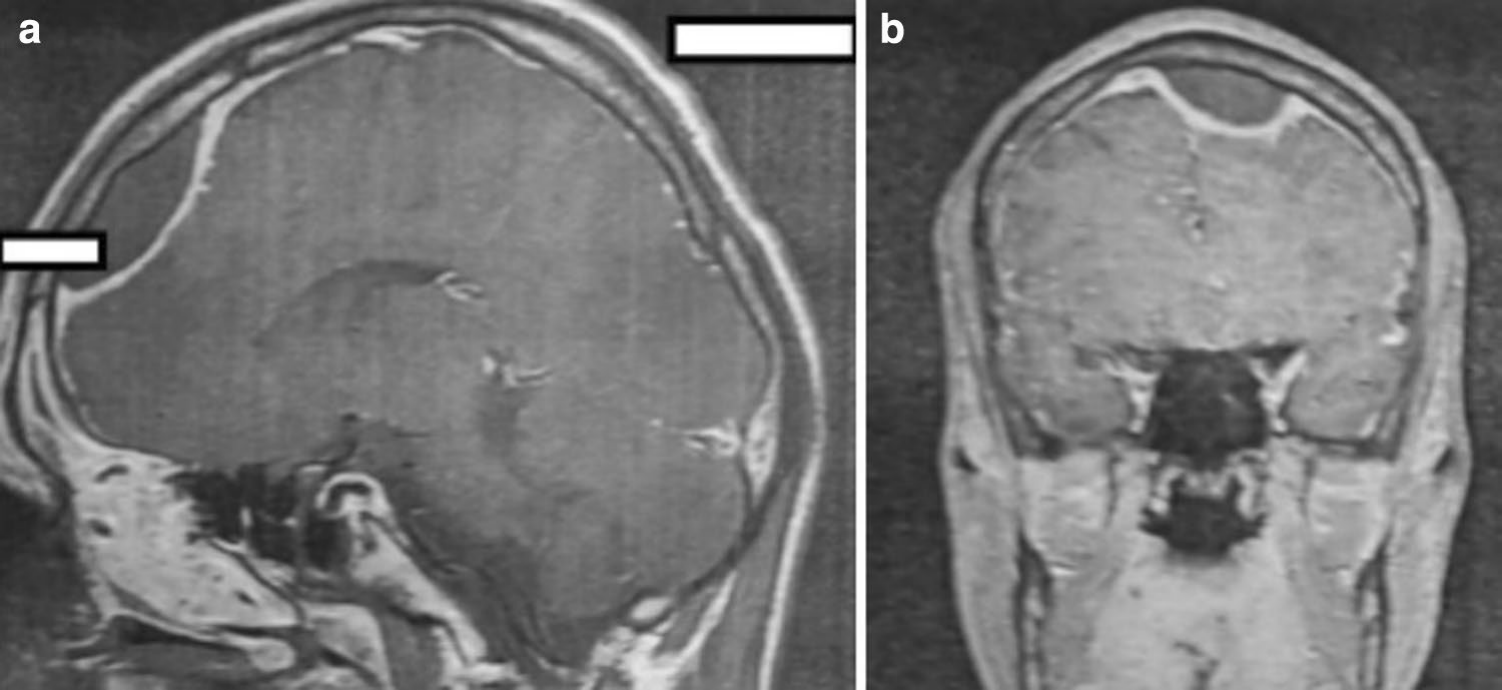
MRI of the cranium in a 20-year-old man. Abscess under the cranium near the left frontal lobe and displacement of the ventricular system to the right

**Fig. 4:**
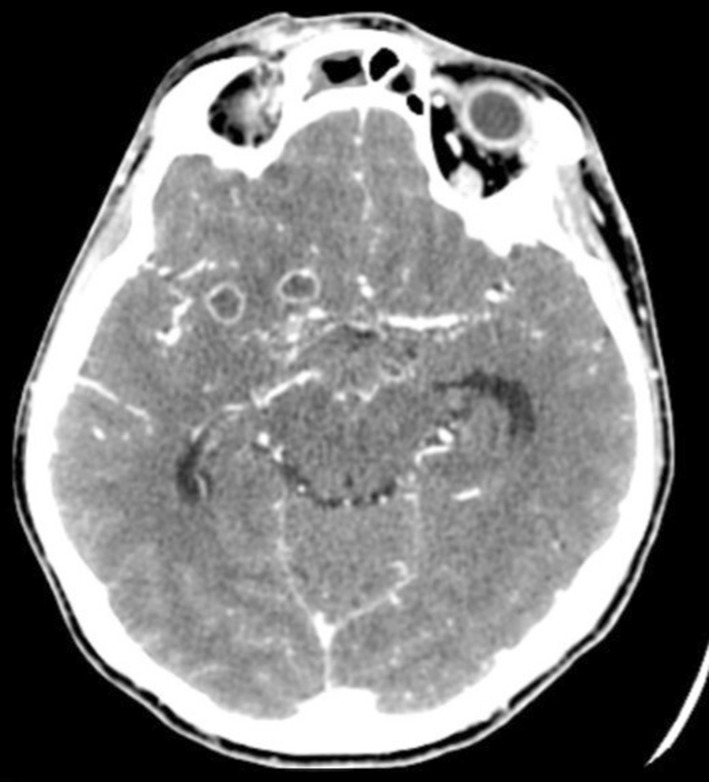
MRI of the cranium in a 28-year-old woman. Two small abscesses with diameters of 8 and 10 mm on the frontal–temporal border on the right side

Most frequently, intracranial complications were associated with abscesses located in the frontal sinus (Figs. 1, 2a, b). In 25 patients, the abscess was on the right side, constituting the most common complication found during all periods (nine in the first period, five in the second and 11 in the third). In a single patient with a left frontal lobe abscess, there were coexisting cranial epidural abscesses and a subdural empyema. In another patient, the frontal abscesses were bilateral. Sixteen patients were diagnosed with an epidural abscess (seven, two and seven in the respective periods), and nine patients were diagnosed with a subdural empyema (three, one and five in the respective periods) (Fig. 3). In a single patient, multiple cerebral abscesses were visualised in the frontal and parietal lobes (Fig. 4). Three patients were admitted to the clinic with symptomatic sinus thrombosis (0, 2 and 1 in the respective periods). Two patients had cerebritis. Fifteen patients had meningitis: eight with diagnosed *Staphylococcus* or *Streptococcus* aetiology and one viral. In two patients, the cerebrospinal fluid was sterile (Tables 2, 3).

**Table 2:** Intracranial complications of sinusitis in specific periods

	1964–1978	1979–1999	2000–2016	Total	*p* value*
Number of patients	16	9	26	51	–
Gender	12 M (75.0%) 4 F (25.0%)	5 M (55.6%) 4 F (44.4%)	19 M (73.1%) 7 F (26.9%)	36 M (70.6%) 15 F (29.4%)	0.8162
Mean age	35	39	39	39	–
Number of complications	24	13	36	73	–
Frontal lobe abscess (including multiple abscesses)	9 (56.3%)	5 (55.6%)	11 (42.3%)	25	0.8394
Epidural abscess	7 (43.8%)	2 (22.2%)	7 (26.9%)	16	0.6815
Subdural empyema	3 (18.8%)	1 (11.1%)	5 (19.2%)	9	0.9738
Multiple intracerebral abscess	0 (0.0%)	1 (11.1%)	2 (7.7%)	3	0.8949
Sinus thrombosis	0 (0.0%)	2 (22.2%)	1 (3.8%)	3	0.3480
Meningitis	3 (18.8%)	2 (22.2%)	10(38.5%)	15	0.3325
Cerebritis	2 (12.5%)	0 (0.0%)	0 (0.0%)	2	0.4489

*Independent Chi-square test between three periods

**Table 3:** Co-occurrence of intracranial complications between 1964 and 2016

	1964–1978	1979–1999	2000–2016	Total
Number of patients	16	9	26	51
Gender (M—male, F—female)	12 M, 4 F	5 M, 4 F	19 M, 7 F	36 M, 15 F
Mean age	35	39	39	39
Number of complications	24	13	36	73
Frontal lobe abscess (including multiple abscesses)	9; 1 was double-sided (1 meningitis)	5; 1 was double-sided (1 subdural empyema)	11 (1 epidural abscess, 1 subdural empyema, 1 cavernous sinusitis, 1 meningitis)	25
Epidural abscess	7 (3 subdural empyema)	2	7 (2 subdural empyema, 1 multiple frontal lobe abscesses, 1 meningitis)	16
Subdural empyema	3 (3 epidural abscess, 1 meningitis, 2 brain tissue inflammation)	1 (1 brain abscess)	5 (2 epidural abscess, 1 multiple frontal lobe abscesses, 4 meningitis)	9
Intracerebral abscess	0	1 (1 meningitis)	2 (1 subdural empyema)	3
Sinus thrombosis	0	2 (1 meningitis)	1 (1 subdural empyema)	3
Meningitis	3 (1 epidural abscess, 1 subdural empyema, 1 frontal lobe abscess)	2 (1 thrombotic sinusitis, 1 multiple intracerebral abscesses)	10 (1 brain abscess, 1 epidural abscess, 4 subdural empyema, 1 thrombotic sinusitis)	15
Cerebritis	2 (2 subdural empyema)	0	0	2

The medical interviews revealed 24 patients (47%) treated for recurrent paranasal sinusitis. Eight of these patients had a previous surgical operation, and of these, four had multiple operations (coexisting aspirin-induced asthma). One patient was hospitalised due to suspicion of an epidural abscess in the course of chronic sinusitis on the right side. Surgical resection was performed; however, the abscess returned to the same location.

In the analysed group, the most common presenting complaint was headaches localised in the frontal and parietal areas (76%), followed by fever (74%) and nausea and vomiting (52%). At the time of admission, 18 patients (35%) were unconscious, 18 (35%) were fatigued, 10 patients (20%) reported a recent epileptic seizure, and 14 (27%) presented with hemiparesis.

The most common presenting symptom in the first period (1964–1978) was headache, followed by nausea and vomiting with frequent loss of consciousness and recurrent severe epileptic seizures. The patient’s condition in most cases was very severe. During the second period (1979–1999), patients mainly reported symptoms of fatigue and stupor and convulsions were less frequently noticed. During the third period (2000–2016), signs of paranasal sinusitis were observed: mainly fever, headaches and nasal congestion. In recent years, symptoms have been predominantly centred around paranasal sinusitis, and neurological signs associated with intracranial complications have been less pronounced.

As far as laryngological signs and symptoms, a deviated nasal septum and tooth decay have been observed in most patients. In seven patients (14%) frontal oedema was noted, 4 (8%) had upper eyelid oedema, and in eight patients (16%) anterior nasal polyps were visualised by way of rhinoscopy. In almost all patients, the nasal mucous membranes were inflamed and red, often presenting with mucosal secretion under the medial conchae. In two patients (4%), acute paranasal sinusitis was accompanied by acute otitis media.

## Treatment

All patients in the analysed group were treated operatively within 12 h of admission to the clinic.

In acute or chronic intracranial complications of sinusitis, the preoperative procedure removed the osteomyelitis. During the first two time periods, sinus surgery was usually performed using an external approach, while in the last period endoscopy and surgical microscopes were used. In the event of serious complications, the frontal sinus was opened through the eyebrow arch and the posterior wall partially removed to displace the dura mater. In the frontal sinuses, (aside from purulent secretions, granulomas, and mucosal hyperplasia) inflammatory lesions in the frontal bone were sometimes encountered. Failure to detect osteomyelitis in the posterior wall did not exclude the presence of epidural and cerebral abscesses or subdural empyemas. The characteristic feature of a sclerotic chalk-white appearance in the posterior wall of the frontal sinus is diagnostic for the presence of the aforementioned abscesses and empyema. Inflammation of the inner layer of the dura matter and epidural abscess often develops where the frontal sinus wall closely adheres to the dura mater.

Since 1964, after discontinuing bone abscess ablation in the paranasal sinuses, direct removal of intracranial complications of sinusitis was initiated. Cerebral abscesses that were superficially located frequently opened spontaneously during the removal of bone lesions in the vicinity of frontal sinuses or ethmoid cells. Another method of treating rhinogenic cerebral abscesses located in the superficial frontal lobe was drainage using a delicate plastic catheter placed into the cavity for a period of 3–5 days, leading to gradual emptying of the abscess.

Most of the patients (25 in total; 9, 5 and 11 in respective periods) were diagnosed with frontal or parietal abscesses and were treated using a closed method. This means the abscess was perforated, and the contents gradually were removed using crystallised penicillin with physiological saline (20,000–30,000 units of crystallised penicillin in 10–20 mL of physiological salt). This method required extreme caution to make sure the pressure in the abscess cavity was regulated and produced the least amount of brain trauma [13]. On average, the replacement of the contents of the abscess had to be performed 2–3 times every 7 days; however, there were cases of full recovery even after one puncture. Since 2007, neuronavigation was used to perforate a cerebral abscess. This allowed for the precise location of the abscess, puncturing of the site and exchange of the contents from the abscess. Neuronavigation was particularly useful in cases where the abscess was distant from the dura of the anterior border of the cranium [7].

Surgical treatment of intracranial complications of sinusitis was preceded by widespread use of empirical antibiotics. During the first period, the recommended treatment was the use of crystalline penicillin and metronidazole. In the last period since 2008, piperacillin, tazobactam and meropenem were used. The purulent discharge from the abscesses was cultured, and microbiological analysis showed mostly Gram-positive bacteria (*Streptococci* and *Staphylococci*). In epidural abscess and subdural empyema, obligate anaerobic strains predominated (*Pseudomonas, Propionibacterium, Bacteroides* and *Fusobacterium*). Only in 30% of cases has there been a correlation between the bacterial flora of the paranasal sinus and abscesses. In roughly 30% of abscesses, the purulent culture was sterile. After the microbiological results returned, targeted antibiotics were used to penetrate the blood–brain barrier. Therapeutic measures continued intravenously three times a day for 4 weeks. Controlled imaging was performed 7–10 days after drainage of the abscess, and a decision regarding further treatment was made on the basis of the imaging result.

## Discussion

Brain tissue is relatively resistant to infection; however, if it spreads, the response is specific to the location of the infection, the type of microorganism and immune system competence [6, 8, 14–16]. Pathogenesis has a significant impact on the oropharyngeal region and leads to disorders of ventilation and drainage of the paranasal sinuses. The most common intracranial complications of sinusitis develop as a result of the inflammation of the frontal sinuses, ethmoid cells, sphenoid sinus and, in rare cases, the maxillary sinuses. Inflammation may spread either by direct contact or continuously through pre-existing openings and canals via bone defects resulting from injuries or incomplete endoscopic procedures in the nose and sinuses as well as imprecisely performed open surgeries [17]. An infection spreading via the blood vessels cannot be ruled out [18]. Abscesses may also develop as infections spread from the diploe, the spongy cancellous bone layer between the cortical bones of the skull and of the anterior table of the frontal sinus, which also play a role in intracranial complications. The extensive, thin-walled vascular network (the veins of Brechet) of the diploe forms a confluent canal of vessels regardless of anatomical bone borders. The veins of Brechet have multiple connections with the veins of the skull, dura mater, frontal bone and cerebral frontal lobes. The thinness of the vessel walls, slower blood flow and close proximity to the mucous membrane of the purulently inflamed sinus all predispose the formation of thrombus infection, which leads to further intracranial complications. The diploe of young males is most prone to infections due to its increased thickness [3, 19].

Intracranial complications of sinusitis can have a wide range of clinical presentations, including frontal or parietal lobe abscesses, meningitis, cerebritis, epidural abscess, subdural empyema, sigmoid sinus thrombosis and frontal bone osteomyelitis with a risk of Pott’s puffy tumour formation.

Paranasal sinus infection with intracranial complications, rapid disease progression, and spreading of inflammation from the paranasal sinuses to the base of the skull all require intensive antibiotic therapy and rhino- or neurosurgery. Depending of the symptoms and state of the patient, many combinations of these methods exist [2, 4]: intensive intravenous antibiotic therapy followed by a neurosurgical procedure to remove the central nervous system abscess and no contemporaneous removal of the paranasal abscess, beginning therapy with the removal of the paranasal sinus abscess followed by antibiotic therapy and neurosurgery. Since 1964, the Otolaryngology Clinic at Poznań University of Medical Sciences has used concomitant intensive antibiotic therapy, surgical paranasal inflammatory lesion removal and treatment of central nervous system abscesses. Antibiotic therapy is initially empirical but is shifted to targeted therapy once the pathogen and its susceptibility is established and lasts 4–6 weeks. Currently, the surgical treatment of paranasal sinuses is most often endoscopic or microscopic; however, in difficult or otherwise justified cases, an external approach that opens the frontal or other paranasal sinuses and a possible exposure of the dura mater of the anterior cranial fossa is used.

The time span of this study allows for a precise analysis of the state of the patients admitted to the clinic. During the first period (1964–1978), 16 patients’ clinical picture was dominated by intense pain in the frontal and parietal areas, with nausea, vomiting with lightheadedness, somnolence, hemiparesis, loss of consciousness and recurrent epileptic episodes. The general state of the patients was poor. During the second and third periods (1979–2016), patients with intracranial complications of sinus infections were more often diagnosed with exacerbations of purulent paranasal sinusitis rather than symptoms of intracranial complications which manifested as increased body temperature and headaches. Up until the patients presented changes in their level of consciousness, epileptic attacks or neurological deficits, their symptoms could have been interpreted as not requiring specialist therapy and hospitalisation. Verification of the symptoms with either CT or MRI allowed for a more precise disease diagnosis.

The following intracranial complications of sinusitis were most common during the presented 53-year-long study: 25 frontal and/or parietal lobe abscesses (34.2%), 16 epidural abscesses (29%), 15 meningitis cases (20.5%), nine subdural empyemas (12.3%), three sigmoid sinus thrombosis cases (4.1%), three small (< 1 cm) intracerebral abscesses (4.1%) and two cerebritis cases (2.7%). These complications were more likely to occur concomitantly, rather than alone. In total, 51 patients presented with 73 complications. It is worth noting that, according to our data, subdural empyema and epidural abscesses usually did not occur spontaneously and most often presented with other intracranial complications. In turn, meningitis occurred concomitantly with other intracranial complications 80% of the time (12/15). Frontal and/or parietal lobe abscesses were the least likely to appear with other complications in only 24% of the cases (6/25) (Table 3).

In 16 patients with epidural abscesses, the lesions were usually located between the posterior bony wall of the frontal sinus and the dura of the anterior cranial fossa and usually showed thickness in granulation tissue. As the abscess expanded, the dura mater of the frontal lobe would separate from the posterior bony wall of the frontal sinus. The epidural abscesses were usually small in size, and among them, five occurred simultaneously with subdural empyema, one with meningitis and one with a frontal lobe abscess. In MRI and CT, they presented as hypodense lens-shaped lesions. The presenting symptoms were uncharacteristic, and the dull headaches and elevated body temperature were initially associated with the inflammation of the frontal (or other) sinus. In these states, meningeal and cerebral symptoms appeared late, sometimes even during subsequent complications. Epidural abscesses were always treated surgically and would usually spontaneously evacuate after exposure of the posterior wall of the frontal sinus.

Subdural empyema (nine total) usually associated with paranasal sinusitis and developed as a result of infection spreading from either the septic thrombophlebitis of the deep paranasal sinus-draining veins of the paranasal mucosa or via direct expansion from osteomyelitis of the posterior wall of the frontal (or other) sinus. These empyemas usually consisted of a suppurative exudate-filled cavity located between the dura and arachnoid maters and, in our study, were among the most life-threatening intracranial complications of sinusitis. In most cases, they coexisted with other intracranial complications: five with epidural abscesses, five with meningitis, two with cerebritis and one with small frontal lobe abscesses. The contact between the inflamed posterior wall of the sinus and the dura mater facilitated the spread of the infection into the subdural space [11]. Subdural empyemas also developed as a result of inflammatory lesions in the frontal and ethmoidal sinuses and from epidural abscesses. An important feature of all nine presented cases of the subdural empyema was the rapidly worsening physical and neurological state of the patient due to the fact that the extensive subdural space does not contain any natural barriers that could limit the spread of infection. However, a reaction to pathogens from the inflammatory focus allowed the subdural space to form adhesions and thus contain the infection. The inflammatory changes associated with subdural empyema usually began in the superior anterior part of one hemisphere and spread posteriorly. It is worth mentioning that subdural empyemas were sometimes located distally to the inflammatory lesion in the wall of the sinuses. The clinical picture was dominated by headaches, elevated body temperature, nausea, vomiting, epileptic attacks, lightheadedness and coma. MRI studies were crucial to their diagnosis, and in CT these abscesses appeared as concave hypodense lesions present above one or both hemispheres or in the space between them.

Lumbar puncture was avoided in the diagnosis or if there was a suspicion of subdural empyema, as it did not deliver valuable information and was associated with the risk of cerebral gomphosis. Neck stiffness was an uncommon sign in patients with a subdural empyema. The treatment, aside from the removal of the sinus-derived bony inflammatory focus, consisted of exposing the dura of the frontal or parietal lobe and locating the pus-filled cavity with the assistance of a neurosurgeon. Treatment options included evacuation of the empyema either by craniopuncture or craniotomy. Intensive antibiotic therapy was continued for approximately 4–6 weeks, and antiepileptic and antiedematous therapies were also applied [20].

Among the 15 cases of sinus-derived meningitis, 5 occurred simultaneously with subdural empyema, 2 with epidural abscesses, 2 with frontal lobe abscesses, 2 with sigmoid sinus thrombosis and 1 with small multiple intracerebral abscesses. The remaining three cases of meningitis did not present with any concomitant complications. The therapy of patients with meningitis consisted of preventative, surgical and general treatments. Preventative treatment focused on keeping the infection from spreading to the subarachnoid space. Inflammation of any sinus could eventually result in meningitis; however, the sphenoidal, frontal and ethmoidal sinuses were most often the cause. The clinical picture was dominated by parietal and spine pain, elevated body temperature and neck stiffness and suppurative rhinitis. In cases where meningitis occurred simultaneously with other intracranial complications, (which occurred most often; 12 total), the course of illness was much less severe. All cases of sinus-derived bacterial meningitis required an MRI to search for any changes in the central nervous system. Patients with meningitis also required the testing C-reactive protein (CRP), leukocytosis and procalcitonin levels, as well as neurological assessments, CT scans and cerebrospinal fluid analysis. Treatment came down to administering directed antibiotic therapy that could cross the brain–blood barrier. If necessary, antiepileptic and antiedematous therapies were also administered. Corticosteroids were only given in life-threatening brain edema and only for short periods of time [2]. The surgical treatment of primary sinus-derived meningitis came down to the complete removal of any focuses of inflammation in the paranasal sinuses.

The clinical picture of the 25 patients with sinus-derived brain abscesses depended on the duration of abscess development, maturity of the capsule and coexistence with any other intracranial complications, including two multiple abscesses, one epidural abscess, two subdural empyemas, one cavernous sinus inflammation case and two cases of meningitis. MRI studies of mature abscesses showed a hyperresonant capsule surrounding a hypodense centre, all of which was surrounded by a hypodense halo of brain oedema. The brain abscesses were described as inflammatory tumour lesions [21, 22]. The clinical symptoms were varied and included mild fevers, vomiting, headaches, personality changes with sudden mood swings and other neurological symptoms. Advanced frontal lobe abscesses were associated with epileptic attacks. It is important to note that in the last 15 years, neurologic symptoms were seen very rarely and, at times, did not present clinically. Treatment consisted of high-dose intravenous antibiotics and neurosurgical abscess drainage and both paranasal and brain abscesses were evacuated during the same procedure. The drainage of frontal and/or parietal lobe abscesses was performed using the so-called closed method [13]. If such an abscess was located deep within the brain tissue, cooperation with a neurosurgeon was a necessity. Intensive antibiotic therapy was continued for at least 4 weeks with the bacteriological study results of the abscess taken into account. Two patients with small multiple sinus-derived frontal lobe abscesses (1 cm) were completely cured after a course of intensive antibiotic therapy.

The analysed material contained three cases of sinus thrombosis, two of which occurred simultaneously with meningitis, and in one case with two small frontal lobe abscesses, suppurative sphenoid, maxillary and ethmoid sinus inflammation was noted. The clinical picture was dominated by severe septic shock with recurrent epileptic attacks. The patient’s condition slowly improved in response to the surgical treatment of the paranasal sinus bone abscess and antibiotic, heparin and intense hydration of the sinus thrombosis.

Analysis of the literature shows that without the administration of antibiotics in intracranial complications of sinusitis, mortality was very high (80–100%) [23–25]. The use of next-generation antibiotics, new imaging methods (MRI and CT), the perfection of sinus surgery techniques (FESS), the development of surgical methods for the base of the skull and the use of neuronavigation all played a significant role in decreasing mortality rates, which are currently between 7 and 15% [3, 6, 8, 9, 26–28]. Data from 1964 to 1978 from our clinic contain three fatal cases, which is 17% of the patients with intracranial complications of sinusitis in that time frame. The deaths could largely be attributed to a delay in both diagnosis and implementation of treatment. The first patient passed away during the 1st day of his stay at the clinic, the second on the 7th day, and the third on the 31st day. The latter two cases, besides the extensive subdural empyema, were also complicated by multiple abscesses in both hemispheres with areas of encephalomalacia. Since 1978, there have been no deaths in the course of intracranial complications of sinusitis; however, eight patients (approximately 15%) developed epilepsy as a consequence of the disease.

In the last 17 years (2000–2016), it has been noted that patients with intracranial complications of sinusitis, aside from those with subdural empyema, have only moderately expressed neurological symptoms, show a more rapid normalisation of biochemical blood markers (leukocytosis, CRP and procalcitonin) and return to health more quickly than patients in earlier years, e.g. 1964–1999. This is especially visible in patients with sinus-derived frontal and/or parietal lobe abscesses.

## Conclusions

1. Simultaneous treatment of acute and chronic inflammatory paranasal sinuses with intracranial complications is an effective and low-burden treatment for patients.
2. Intracranial complications of sinusitis often coexist with other complications of this type.
3. In our study, intracranial complications of sinusitis are most commonly found to be abscesses in the frontal or parietal lobes followed by epidural abscesses and meningitis.
4. Coexistence of two or more intracranial complications significantly worsens the overall prognosis of the patient’s health and is a direct threat to his/her life.
5. In the last 17 years, neurological symptoms have been poorly characterised in patients with intracranial complications of sinusitis, particularly with a frontal lobe abscess.
6. Since 1978, the Department of Otolaryngology has experienced no deaths due to treatment of intracranial complications of sinusitis.
7. In acute and chronic paranasal sinusitis, a high degree of suspicion should be maintained due to the possibility of intracranial complications.
